# Traditional Brazilian Diet and Olive Oil Reduce Cardiometabolic Risk Factors in Severely Obese Individuals: A Randomized Trial

**DOI:** 10.3390/nu12051413

**Published:** 2020-05-14

**Authors:** Annelisa Silva e Alves de Carvalho Santos, Ana Paula dos Santos Rodrigues, Lorena Pereira de Souza Rosa, Matias Noll, Erika Aparecida Silveira

**Affiliations:** 1 Programa de Pós-Graduação em Ciências da Saúde, Faculdade de Medicina, Universidade Federal de Goiás, Goiânia 74.650-050, GO, Brazil; anapsr@gmail.com (A.P.d.S.R.); lorenapsrosa@yahoo.com.br (L.P.d.S.R.); matiasnoll@yahoo.com.br (M.N.); 2 Instituto Federal Goiano, Rodovia Go-154, Km 03, s/n, Ceres 76300-000, GO, Brazil

**Keywords:** extra virgin olive oil, cholesterol, dietary pattern, morbid obesity, dietary intervention

## Abstract

Cardioprotective effects associated with extra virgin olive oil (EVOO) have been studied within the Mediterranean diet. However, little is known about its consumption in the traditional Brazilian diet (DieTBra) or without any dietary prescription, particularly in severely obese individuals. This study aimed to assess the effectiveness of DieTBra and EVOO in cardiometabolic risk factor (CMRF) reduction in severely obese individuals. We conducted a parallel randomized clinical trial with 149 severely obese individuals (body mass index ≥ 35.0 kg/m^2^) aged 18–65 years, assigned to three groups: 52 mL/day of EVOO (*n* = 50), DieTBra (*n* = 49), and DieTBra + 52 mL/day of EVOO (*n* = 50). Participants were followed up for 12 weeks. Low-density lipoprotein cholesterol (LDL-c) was the primary endpoint and several cardiometabolic parameters were secondary endpoints. Endpoints were compared at baseline and at the end of the study using analysis of variance, the Kruskal–Wallis test, and Student’s *t*-test. The TC/High-density lipoprotein (HDL) ratio (−0.33 ± 0.68, *p* = 0.002) and LDL/HDL ratio (−0.26 ± 0.59, *p* = 0.005) decreased in the EVOO group. Delta values for all variables showed no significant statistical difference between groups. However, we highlight the clinical significance of LDL-c reduction in the EVOO group by 5.11 ± 21.79 mg/dL and in the DieTBra group by 4.27 ± 23.84 mg/dL. We also found a mean reduction of around 10% for Castelli II (LDL/HDL) and homocysteine in the EVOO group and TG and the TG/HDL ratio in the DieTBra group. EVOO or DieTBra when administered alone lead to reduction in some cardiometabolic risk parameters in severely obese individuals.

## 1. Introduction

In the past few years, severe obesity prevalence has increased more than class I obesity, followed by a higher risk of morbidity and mortality [1]. Accessible and non-invasive treatments to reduce cardiometabolic risk factors (CMRF) are needed and relevant. In Brazil, between 2006 and 2013, severe obesity increased by 36.4%, revealing a worrying epidemiological scenario [2]. The few interventional studies focused on CMRF reduction in severely obese individuals mainly analyzed physical activity programs with low-calorie or low-fat diets to promote weight loss [3,4,5]. Although it is relevant to evaluate the effectiveness of healthy dietary patterns or plant-based oils on CMRF reduction in severely obese individuals, research on this matter is still scarce.

The Mediterranean diet (MedDiet) plays an important role in cardiometabolic risk reduction, mostly because of the large amounts of olive oil [6,7,8,9]. In moderate quantities, extra virgin olive oil (EVOO) consumption is associated with a reduction of certain cardiovascular disease outcomes [10]. The health benefits and cardioprotective effects associated with EVOO consumption have been studied within the MedDiet in several cohort studies in Europe [11], where it’s use is common.

However, little is known about incorporating olive oil into other healthy dietary patterns or whether introducing olive oil without any dietary modifications can reduce cardiometabolic risk factors [11], mainly in randomized clinical trials (RCT). Both MedDiet and the traditional Brazilian diet (DieTBra) are plant-based healthy dietary patterns. DieTBra has food components available in several countries, which makes it feasible. DieTBra is characterized by rice and beans consumed in main meals (lunch and dinner) along with small portions of red meat, raw and cooked vegetables, dairy products in small meals, bread, and fruits. Additionally, DieTBra includes rare seafood, nuts, wine, and olive oil consumption compared to the MedDiet. The predominant oil used in DietBra to prepare meals is soy or corn oil [12,13].

Studying the effect of EVOO and another healthy dietary pattern independent of the MedDiet as a new treatment approach to reduce cardiometabolic risk in severely obese individuals is relevant, considering the high cardiometabolic risk usually presented by this population and the lack of studies regarding the cardiovascular effects of the DieTBra. Therefore, the aim of this study was to evaluate whether DieTBra, EVOO, or a combination of DieTBra with EVOO can reduce CMRFs in severely obese individuals.

## 2. Materials and Methods

### 2.1. Study Design and Ethical Statements

This study is a randomized clinical trial with parallel design conducted with severely obese individuals in Midwest Brazil between June 2015 and February 2016. The study is part of a larger randomized clinical trial entitled, “Effect of Nutritional Intervention and Olive Oil in Severe Obesity-DieTBra Trial,” registered at ClinicalTrials.gov (NCT02463435). The primary endpoints were changes in anthropometric and body composition measurements, results that were partially reported elsewhere [14,15]. The endpoint of interest in the present study was the change in metabolic parameters related to cardiometabolic risk, as detailed in the topics 4.8 and 4.10. The study protocol was approved by the Clinical Hospital’s Ethics Committee of the Federal University of Goiás (protocol no. 747,792). All participants who agreed to engage in this study provided informed consent. The investigation was conducted in accordance with the principles outlined in the Declaration of Helsinki.

### 2.2. Participants

Participants were between the ages of 18 and 65 years, had a body mass index (BMI) ≥35 kg/m^2^, and resided in Goiânia and/or the metropolitan region. The exclusion criteria were a history of bariatric surgery, weight loss ≥8% in the past three months [16], nutritional treatment in the past two years, pregnancy and lactation, allergies, intolerance to any vegetable oil, and people with special needs who were not able to walk, hear, or speak. Individuals with HIV/AIDS, cardiac insufficiency, liver or kidney failure, chronic obstructive pulmonary disease, cancer that required treatment, and daily use of anti-inflammatory drugs and corticosteroids were also excluded from the study sample.

### 2.3. Baseline

**Figure 1:**
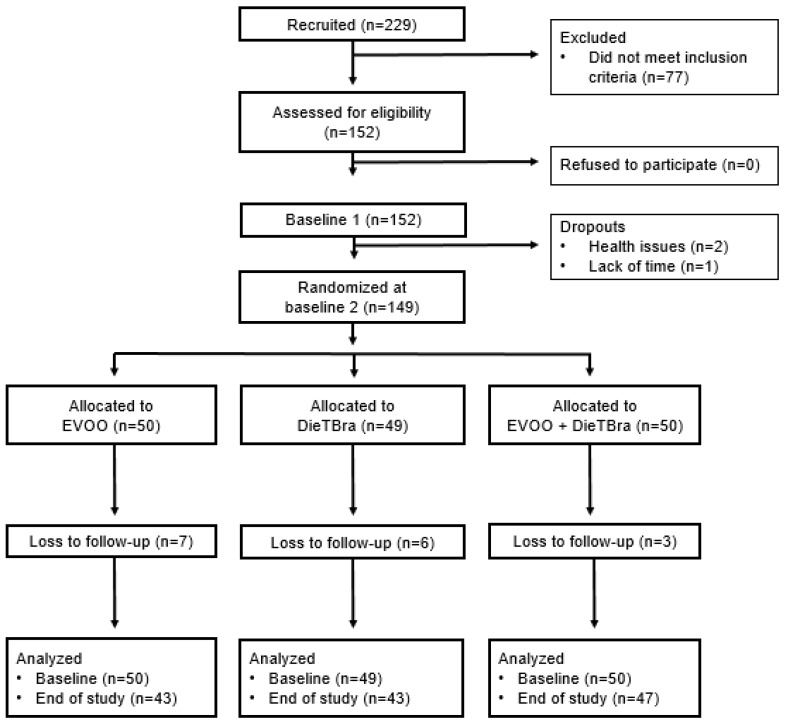
Flow diagram of enrollment, randomization, and follow-up. DieTBra: traditional Brazilian diet. EVOO: extra virgin olive oil (*n* = 149).

Severely obese individuals were recruited from the Nutrition in Severe Obesity Outpatient Clinic of the Clinical Hospital of the Universidade Federal de Goiás (UFG). There were 229 people recruited and after screening for eligibility, 152 participants met the inclusion criteria and provided informed consent (Figure 1).

Baseline data were conducted in two stages within a week. In the first stage, sociodemographic and anthropometric variables were collected through a structured questionnaire and an accelerometer device was placed on the participants. Participants were told to return in one week for the second baseline stage, during which blood collection and bioimpedance analysis, accelerometer device removal, and consultation with the registered dietitian were performed. During this consultation, participants received the assigned interventions and were instructed to return in four weeks for follow-up visits.

### 2.4. Randomization

A randomization list was generated online before data collection commencement (www.randomization.com). Participants were allocated to one of three interventions groups in a 1:1:1 ratio after baseline procedures were completed. They were then assigned colors allowing the researchers to identify which group they belonged to during follow-up visits. The randomization was performed by the same trained researcher during the entire study, in a separate room.

### 2.5. Interventions

There were three intervention groups: (1) EVOO; (2) DieTBra; and (3) DieTBra + EVOO. The participants assigned to the first group received EVOO with <0.2% acidity, cold pressed and packed in photosensitive sachets with a capacity for 13 mL. The individual daily serving size was 52 mL (4 sachets). No nutritional counseling, dietary prescriptions, or recommendations for regular physical activity were provided to this group. The participants were instructed to consume the EVOO sachets either alone, along with principal meals (lunch and dinner), in salads, or other preparations of their preference. Olive oil was to be consumed unheated and at room temperature. EVOO was purchased from a reputable company, following rigorous quality standards, through funding granted to the larger study. An independent quality analysis was conducted to evaluate ash content, acidity, and purity of the EVOO used in our study, along with the fatty acid profile (data not shown).

DieTBra was widely consumed before the nutritional transition. Over the years, unhealthy dietary habits typical of the Western diet such as fast and ultra-processed foods have been incorporated [17]. Nutritional intervention based on DieTBra would restore healthy diets featured by adequate intake of vitamins, minerals, dietary fiber, and the consumption of fresh and minimally processed foods [18]. A comparative box with the main features of both DieTBra and MedDiet is available as Supplementary Table S1.

Both groups assigned DieTBra were prescribed individualized eating plans. During the intervention period, the term “healthy eating plan” was used over “diet,” since the latter carries a negative connotation [19]. Participants in the DieTBra and DieTBra + EVOO groups were advised to have 4–6 meals/day, to consume fruits and vegetables daily as well as the combination of rice and beans in the principal meals, to prefer whole foods over refined foods, to have adequate water intake, and to avoid ultra-processed foods [18]. In addition, participants assigned to the DieTBra and DieTBra + EVOO groups were encouraged to practice at least 150 min of moderate-to-vigorous physical activity per week [20].

The total energy value (TEV) of the prescribed eating plan was based on resting energy expenditure (REE), total energy expenditure (TEE), and weight loss goals. REE was calculated according to an equation developed for severely obese individuals, considering the fat free mass [21] assessed by multifrequency bioimpedance. The total energy expenditure (TEE) was calculated considering REE, physical activity factor, and thermal effect of food, the latter standardized as 8% of TEE [22]. The physical activity factor was based on the level of physical activity assessed by the Global Physical Activity Questionnaire Version 2 [23]. A reduction of 500 to 1100 kcal from TEE was set, aiming at weight loss of 0.5 kg to 1 kg per week according to individual weight loss goals established for different BMI ranges. This was determined as a percentage change from baseline body weight: 5% (35.00–37.50 kg/m^2^); 6% (37.51–40.00 kg/m^2^); 7% (40.01–45.00 kg/m^2^); 8% (45.01–50.00 kg/m^2^); 9% (50.01–55.00 kg/m^2^); and 10% (>55.01 kg/m^2^).

The DieTBra + EVOO group received both interventions (DieTBra + 52 mL of olive oil/day, with slight differences in macronutrient distribution from the DieTBra group). The macronutrient distribution range within the TEV in the DieTBra group was 50% carbohydrates, 20% protein, and 30% lipids. The DieTBra + olive oil group ranged from 35–40% carbohydrates, 10–15% protein, and 45–55% lipids. This difference occurred because of the fat content of olive oil, mainly composed of mono-unsaturated fatty acids (MUFA). To guarantee isocaloric TEV of the prescribed eating plans in both groups, the food portions were reduced to include 470 kcal from 52 mL of olive oil in the DieTBra + EVOO group.

### 2.6. Blinding

Considering the complexity of nutritional interventions as behavioral exposures, blinding of dietary interventions in RCT are often difficult and impractical [24,25]. In this clinical trial, the blinding was assured in the biochemical analysis. Logistically, the study was designed to not allow participants from different groups to have contact with each other. The EVOO sachet was adequately prepared according to the recommendations of the National Health Surveillance Agency of Brazil for Clinical Trials to mask this intervention. During the study period, all members of the research team were instructed to define the sachet as a “dietary supplement enriched with bioactive compounds” or just “nutritional supplement” instead of the term “olive oil”.

### 2.7. Follow-Up

After baseline procedures and randomization, the participants returned every four weeks, for a total of 12 weeks.

### 2.8. Study Variables

All variables used in this study were properly registered in a structured questionnaire. Biochemical markers related to cardiometabolic risk were assessed at baseline and at the end of follow-up with 12 h fasting including blood glucose, insulinemia, hemoglobin A1c (HbA1c), homeostatic model assessment of insulin resistance (HOMA-IR), total cholesterol (TC), low-density lipoprotein cholesterol (LDL-c), high-density lipoprotein cholesterol (HDL-c), triglycerides (TG), and homocysteine. Non-HDL-c was calculated as the difference between TC and HDL-c. Three atherogenic indexes were also evaluated: triglycerides-to-HDL ratio (TG/HDL), Castelli I (TC/HDL), and Castelli II (LDL/HDL). All biochemical examinations were performed according to standardized procedures: enzymatic colorimetric (fasting blood glucose, TC, LDL-c, HDL-c, and TG); electrochemiluminescence (fasting insulinemia, HOMA-IR, and homocysteine); and immunoturbidimetry (HbA1c). The blood samples were analyzed at the Rômulo Rocha Laboratory of the Universidade Federal de Goiás, which has an excellence certification on the quality conferred by the National Quality Control Program from the Brazilian Society of Clinical Analyses.

Systolic and diastolic blood pressures (BPs) were evaluated using an automatic blood pressure monitor with an adequate cuff for obese individuals (Omrom HEM-742INT). Arterial BP measurement was performed after the participant rested for 20 min in a seated position. Two BP measures within 2–3 min intervals were taken on each visit, and the mean value was calculated [26].

Sedentary behavior (SB) was defined as participation in activities that do not significantly increase energy expenditure, such as sitting and reclining during walking hours [27]. In our study, SB was objectively measured with a wrist-worn 30 Hz frequency triaxial accelerometer (ActiGraph wGT3X), used on six consecutive days, including two weekend days, 24 h/day. The accelerometer was placed on the non-dominant wrist both at baseline and the end of follow-up. Raw data from the accelerometer were expressed in gravitational equivalent units, namely, milligravities (mg), or simply milli-g, being 1000 mg = 1 g = 9.81 m/s^2^. Registered activities with mean acceleration lower than 50 mg were classified as SB, originally measured in mean minutes spent per day in SB [28]. For the purpose of this analysis, time spent in SB was converted to h/day, without differentiating weekdays and weekend days. All accelerometer data were downloaded to the Actilife v. 6.11.7 software and exported to Stata 12.0.

Participants’ body weight and height were measured for BMI calculation (weight in kg/height in m^2^). An electronic digital scale with 200 kg capacity and 100 g precision was used for weight measurement (Welmy), with the patient barefoot and wearing light clothes, without any objects in their pockets. Height was measured to the nearest 0.1 cm with a stadiometer coupled with the electronic digital scale. Body fat percentage was measured through multifrequency bioelectrical impedance (InBody S10).

Dietary intake was estimated through six 24 h recalls, applied by a trained nutritionist. After data collection, the participants’ dietary intake information was input into nutrition software (AVANUTRI) to estimate the total energy value and macronutrient distribution range of each 24 h recall. We considered the mean of three 24 h recalls at baseline and another three at the end of the study to estimate changes in calories and macronutrient intake during 12 weeks of follow-up. The results of changes in calories and macronutrient intake are available in the Supplementary Table S1.

### 2.9. Research Team and Quality Control

Standard operating procedures (SOPs) were developed by the researchers to systematize the practice and procedures in all study stages. Registered dietitians received specific training to assess participants’ motivation and standardize the treatment protocols and approaches. Periodic training and group meetings were conducted to ensure that procedure standardization was aimed at quality data collection. One team member was responsible for reminding participants of their appointments via telephone calls. This strategy was used to enhance patient compliance with the study schedule. At each appointment, participants were asked to bring in empty sachets of the dietary supplement, as well as those not consumed, to evaluate compliance and amount of EVOO consumed during the period.

### 2.10. Statistical Analysis, Endpoints, and Sample Size

Data were entered by two independent researchers using EpiData 3.1 software to check for posterior inconsistencies. Stata 12.0 statistical package was used for data analysis. Normality distribution for continuous variables was assessed using the Kolmogorov–Smirnov test. Intention-to-treat analysis was performed.

As this is a secondary analysis of the major clinical trial, the primary endpoint of interest in this study was a change in cardiometabolic risk assessed through LDL-c levels. All other variables previously described were secondary endpoints. BMI was used for participant characterization at baseline only.

Comparison between groups was performed for the mean values at baseline, at the end of follow-up, and for delta (∆) mean values. Delta values were calculated as the 12-week value minus the baseline value. A comparison of mean values at baseline and at the end of follow-up for each intervention group was also carried out.

Analysis of variance (ANOVA), the Kruskal–Wallis test, and Student’s *t*-test (paired and unpaired) were performed. Bonferroni correction was used to test pair differences in weight change. A graphical analysis was conducted to identify outliers (data not shown). All criteria for analysis of covariance (ANCOVA) were tested, but none of the endpoint measures and potential confounders (∆ body weight and ∆ sedentary behavior) met the criteria [29]. Statistical significance was set at *p* < 0.05 for all analyses.

The sample size estimate was performed based on the central limit theorem. According to this theorem, a sample with a size equal to or greater than 30 tends to present normality in the distribution of means and it is also enough to find significant differences [30]. Therefore, a target of attracting 50 individuals was established for each arm of the study considering a safety margin.

## 3. Results

Between baseline stages 1 and 2, three individuals dropped out of the study due to health issues or lack of time to participate in the study. Hence, 149 participants were randomized into the three intervention groups: 50 participants in the EVOO group, 49 in the DieTBra group, and 50 in the DieTBra + EVOO group. Sixteen participants were lost to follow-up during the study, resulting in a dropout rate of 10.7%. At the end of 12 weeks, 43 participants remained in the EVOO group, 43 in the DieTBra and 47 in the DieTBra + EVOO (Figure 1). No adverse effects from EVOO intake were reported.

Study participants were aged 39.63 ± 8.82 years and 85.23% were females. Approximately 35% of study participants had LDL-c values ≥120 mg/dL at baseline, with no statistical difference between the intervention groups (*p* = 0.430). No differences were found for age, BMI, or other primary and secondary endpoints at the beginning of the study (*p* > 0.05), indicating groups’ homogeneity after randomization (Table 1).

Comparing primary and secondary endpoints at baseline and at the end of follow-up, we found few statistically significant results. Castelli I (TC/HDL) (*p* = 0.002) and Castelli II indices (LDL/HDL) (*p* = 0.005) showed a statistically significant decrease in the EVOO group. In the DieTBra + EVOO group, diastolic BP (0.053) and systolic BP (*p* = 0.072) decreased, although without statistical significance. Non-HDL-c levels (0.069) in the EVOO group decreased, however non-significantly (Table 2). All of these results were found in paired analysis. Unpaired analysis was also performed, but no statistical differences were found.

The mean body weight changes and the respective 95% confidence intervals (CIs) in all groups were as follows: EVOO 1.66 ± 2.94 kg (95% CI: 0.76–2.57 kg); DieTBra −2.65 ± 5.53 kg (95% CI: −4.36–0.95 kg); and DieTBra + EVOO −1.64 ± 3.47 (95% CI: −2.66–0.62 kg). Regarding the mean body weight change, statistically significant differences were observed only for EVOO versus DieTBra (*p* < 0.001) and EVOO versus DieTBra + EVOO (*p* = 0.001) groups. As for body fat percentage, there was a slight reduction in all intervention groups, although no statistically significant differences within and between groups were found (EVOO: −0.83 ± 3.82, 95% CI −2.04–0.37; DieTBra: −0.49 ± 4.01, 95% CI −1.74–0.76; and DieTBra + EVOO: −0.32 ± 1.50, 95% CI −1.77–0.33).

Delta values for all variables showed no significant statistical difference between groups (Table 3), even when outlier values were excluded from analysis (data not shown). Although not statistically significant, is important to show that LDL-c reduced by 5.11 ± 21.79 mg/dL in the EVOO group and by 4.27 ± 23.84 mg/dL in the DieTBra group. We observed a mean reduction of around 10% for Castelli II (LDL/HDL) and homocysteine in the EVOO group and TG and the TG/HDL ratio in the DieTBra group (Table 3). Delta values were also analyzed as two-by-two comparisons (EVOO versus DieTBra, EVOO versus DieTBra + EVOO, and DieTBra versus DieTBra + EVOO), but no statistical difference was found (data not shown).

Caloric and macronutrient intake at baseline and at the end of follow-up are disposed in Table 4.

## 4. Discussion

As far as we know, this is the first study assessing the effectiveness of the traditional Brazilian diet and extra virgin olive oil on cardiometabolic risk factors in severely obese individuals. Our study provided positive results for consumption of olive oil in normal or DieTBra-based healthy diets, as evidenced by the reduction in cardiometabolic risk factors in this study. This study adds important contributions in both the field of nutrition and the field of cardiology because it demonstrates the important role of nutritional interventions in cardiometabolic risk factor prevention in severely obese individuals.

A reduction of around 5 mg/dL in LDL-c from baseline was found in both the EVOO and DieTBra groups after 12 weeks. According to the American Association of Clinical Endocrinologists and American College of Endocrinology Guidelines, several lipid-lowering drug classes evaluated in different follow-up periods reduced LDL-c by 10–70% [31]. Compared to lipid-lowering medications, the 5% reduction in LDL-c found in our study was modest. However, neither dietary intervention had adverse effects, and both are more cost-effective than medication, allowing for feasible CMRF management strategies.

Our study is also comparable to other nutritional interventions that reduce LDL-c. One recent review showed that currently available supplements and functional foods, either alone or in combination, effectively reduced LDL-c by approximately 5% to 25% [32]. Consumption of water-soluble, viscous-forming fibers reduced LDL-c levels by about 5–10% [33], and phytosterols consumption reduced LDL-c by 13 mg/dL [34]. In summary, many nutritional interventions can reduce LDL-c, and more pronounced effects could be associated with higher baseline LDL-c concentrations [34].

LDL-c oxidation is an early event in atherosclerosis development and the essential cause of coronary heart disease (CHD) [35]. Individuals should be informed early about the risks of dyslipidemia to better understand the benefits of lifestyle modifications [36]. Diets including significant amounts of plant-based foods including fruits, vegetables, nuts, seeds, and legumes and less animal-derived and processed foods assist in the prevention of cardiovascular disease [37]. Consequently, it is very important to promote the traditional Brazilian diet as a nutritional prevention strategy. Nutritional strategies aimed at reducing LDL-c levels, such as moderate EVOO consumption and minimally processed diets as demonstrated here, agree with existing evidence for cardiovascular health nutrition recommendations [10,38].

A significant reduction in the Castelli I (TC/HDL) and Castelli II (LDL/HDL) indices was observed in the EVOO group between baseline and week 12. This result can be explained by the combined influence of MUFA and polyphenols in olive oil on HDL-c function [39], stimulating macrophage-specific reverse cholesterol transport [40].

Although we did not observe statistically significant results in the comparison of delta values between groups for the cardiometabolic risk factors, our results are clinically significant. Castelli II index (LDL/HDL) and homocysteine levels were reduced by 11% and 15% in the EVOO group; TG and TG/HDL ratios fell about 9% in the DieTBra group; and HOMA-IR were reduced by approximately 9% in the DieTBra + EVOO group. The lack of statistical significance may be due to similar reductions observed in all groups. To comply with ethical standards, all severely obese individuals received at least one type of nutritional intervention in our study.

Individuals in the DieTBra and EVOO + DieTBra groups were instructed to exercise for at least 150 min a week. The EVOO group did not receive any recommendations. Despite that, physical activity levels did not change during the study, either for moderate to vigorous physical activity or for sedentary behavior in the three groups. It is important to note that both at baseline and at the end of the trial, individuals did not reach the minimum of 150 min of physical activity recommended by the World Health Organization.

Considering the changes during 12 weeks of intervention, the EVOO and DieTBra groups apparently had better results than the DieTBra + EVOO group, although the results were not statistically significant. At first, we hypothesized that the DieTBra + EVOO group would have better results. However, the simultaneity of two nutritional interventions seems to be a challenge in treating people with severe obesity, and this could be a limitation of our study. The amount of food consumed in the DieTBra + EVOO group was lower than the DieTBra group alone, including the 470 kcal ingested from EVOO. Meal portions were adapted to achieve similar caloric amounts in both groups according to the weight loss target. This could have influenced the treatment’s adherence in the DieTBra + EVOO group, considering severely obese individuals usually consume larger meals [41].

As the limitations of this kind of study imply behavior changes such as adherence to a nutritional intervention, W. Willet argued how complicated or impossible it is to include blinding and to monitor real adherence in these studies [42]. Clinical studies with dietary interventions depend on adherence, real food consumption, and food reporting, despite the fact that none of the participants are in a controlled setting like a laboratory. Another possible limitation could be the difference in macronutrient prescription between groups. DieTBra + EVOO had a higher fat percentage prescription than the DieTBra group but were equal in terms of kilocalories. This difference was not significant between groups at the end of follow-up or as the average of carbohydrate and protein percentages, which were close to the dietary reference intakes ranges [43]. The third limitation could be the lack of a control group without any intervention. However, Brazilian Research Ethics Committees consider it unethical to prevent individuals with health issues from accessing treatment. Despite the limitations mentioned above, we emphasize some strengths of our study, such as the sample size, the strong adherence to follow up, multiple statistical comparisons, and significant results. We also highlight the methodological rigor in all steps of this research, which contributed to the quality of the data in this clinical trial.

Our study revealed that a nutritional intervention based either on DieTBra or extra virgin olive oil has beneficial effects in cardiometabolic parameters in severely obese individuals, considering the clinical significance of the results. DieTBra has some advantages over MedDiet, such as food accessibility and availability in other world regions including Latin America and Asia, and adaptability to cultural eating habits worldwide. The main components of DieTBra including rice, beans, small portion of meats, and local fruits and vegetables, are affordable and easy to obtain worldwide.

Considering that severe obesity is increasing exponentially all over the world, the development of nutritional interventions aimed at reducing CMRF in this population is critical. Non-invasive treatment studies with severely obese individuals are scarce in the literature. Consequently, further research is necessary to help create and revise public policies that target severe obesity and consequently improve clinical treatment protocols regarding CMRF reduction.

## 5. Conclusions

In conclusion, switching to a plant-based traditional Brazilian diet with individualized counseling and adding EVOO into the usual diet is a promising way of reducing important cardiometabolic risk factors in individuals with severe obesity.

## Figures and Tables

**Table 1:** Baseline characteristics according to randomization group.

Variables	Total *n* = 149	EVOO *n* = 50	DieTBra *n* = 49	DieTBra + EVOO *n* = 50
	Mean ± SD	Mean ± SD	Mean ± SD	Mean ± SD
Age, years	39.63 ± 8.82	38.00 ± 8.00	39.00 ± 8.00	42.00 ± 10.00
BMI, kg/m^2^	45.77 ± 6.41	45.52 ± 6.35	46.03 ± 6.20	45.78 ± 6.77
LDL-c, mg/dL	109.44 ± 35.48	106.94 ± 29.96	106.40 ± 32.56	114.76 ± 42.51
Fasting blood glucose, mg/dL	109.95 ± 45.24	104.44 ± 29.02	107.43 ± 35.15	117.94 ± 63.45
Systolic BP, mmHg	128.19 ± 17.88	127.59 ± 16.97	126.60 ± 17.86	130.36 ± 18.89
Diastolic BP, mmHg	85.65 ± 13.65	86.58 ± 17.84	84.34 ± 11.50	86.01 ± 10.58
Fasting insulinemia, µUI/mL	23.42 ± 14.86	25.24 ± 17.74	24.40 ± 14.63	20.65 ± 11.39
Hemoglobin A1c, %	6.30 ± 1.43	6.13 ± 1.27	6.26 ± 1.42	6.52 ± 1.58
HOMA-IR	6.40 ± 4.89	6.78 ± 5.87	6.68 ± 4.76	5.75 ± 3.87
Total cholesterol, mg/dL	189.12 ± 38.10	187.74 ± 37.59	184.31 ± 33.40	195.22 ± 42.61
HDL-c, mg/dL	47.62 ± 11.35	47.18 ± 10.32	48.45 ± 10.51	47.26 ± 13.16
Triglycerides, mg/dL	160.31 ± 78.40	160.46 ± 79.85	154.75 ± 87.76	165.60 ± 67.72
Non-HDL-c, mg/dL	141.49 ± 37.79	140.56 ± 36.73	135.86 ± 32.91	147.96 ± 42.74
TG/HDL Ratio	3.58 ± 2.12	3.61 ± 2.14	3.38 ± 2.19	3.76 ± 2.04
Castelli I Index (TC/HDL)	4.13 ± 1.13	4.13 ± 1.13	3.93 ± 0.93	4.33 ± 1.27
Castelli II Index (LDL/HDL)	2.42 ± 0.96	2.37 ± 0.88	2.29 ± 0.81	2.58 ± 1.15
Homocysteine, mmol/L	9.80 ± 8.36	9.91 ± 12.78	9.12 ± 3.84	10.36 ± 5.68
Sedentary behavior, h/d	19.61 ± 1.39	19.52 ± 1.22	19.52 ± 1.36	19.79 ± 1.57

EVOO: extra virgin olive oil. DieTBra: traditional Brazilian diet. BMI: body mass index. LDL-c: low-density lipoprotein cholesterol. BP: blood pressure. HOMA-IR: homeostatic model assessment of insulin resistance. HDL-c: high-density lipoprotein cholesterol. TG: triglycerides. TC: total cholesterol.

**Table 2:** Comparison of mean values at baseline and at the end of follow-up for cardiometabolic risk factors in severely obese individuals according to each intervention group.

Endpoints	EVOO			DieTBra			DieTBra + EVOO		
	Baseline (*n* = 50)	12 Weeks (*n* = 43)	*p* ^a^	Baseline (*n* = 49)	12 Weeks (*n* = 43)	*p* ^a^	Baseline (*n* = 50)	12 Weeks (*n* = 47)	*p* ^a^
LDL-c, mg/dL	106.94 ± 29.96	101.30 ± 29.76	0.131	106.40 ± 32.56	100.72 ± 32.55	0.258	114.76 ± 42.51	114.52 ± 30.30	0.698
Fasting blood glucose, mg/dL	104.44 ± 29.02	100.24 ± 21.39	0.651	107.43 ± 35.15	101.07 ± 25.57	0.129	117.94 ± 63.45	116.02 ± 55.12	0.505
Systolic BP, mmHg	127.59 ± 16.97	126.67 ± 16.43	0.806	126.60 ± 17.86	124.63 ± 15.40	0.171	130.36 ± 18.89	126.60 ± 13.31	0.072
Diastolic BP, mmHg	86.58 ± 17.84	83.91 ± 11.11	0.724	84.34 ± 11.50	82.33 ± 10.59	0.150	86.01 ± 10.58	83.50 ± 9.68	0.053
Fasting insulinemia, µUI/mL	25.24 ± 17.74	25.24 ± 11.67	0.927	24.40 ± 14.63	25.38 ± 12.96	0.747	20.65 ± 11.39	21.41 ± 12.13	0.505
Hemoglobin A1c, %	6.13 ± 1.27	6.09 ± 0.93	0.488	6.26 ± 1.42	6.08 ± 1.22	0.174	6.52 ± 1.58	6.51 ± 1.72	0.912
HOMA-IR	6.78 ± 5.87	6.38 ± 3.61	0.711	6.68 ± 4.76	6.53 ± 4.00	0.728	5.75 ± 3.87	6.26 ± 6.33	0.428
Total cholesterol, mg/dL	187.74 ± 37.59	180.69 ± 35.79	0.242	184.31 ± 33.40	178.28 ± 35.45	0.137	195.22 ± 42.61	195.06 ± 33.41	0.794
HDL-c, mg/dL	47.18 ± 10.32	47.18 ± 49.21	0.133	48.45 ± 10.51	48.45 ± 48.98	0.734	47.26 ± 13.16	49.51 ± 10.48	0.197
Non-HDL-c, mg/dL	138.91 ± 33.32	131.48 ± 32.24	0.069	136.32 ± 33.61	129.30 ± 32.62	0.081	148.55 ± 42.54	145.55 ± 34.13	0.566
Triglycerides, mg/dL	160.46 ± 79.85	151.35 ± 65.53	0.296	154.75 ± 87.76	142.46 ± 64.96	0.069	165.60 ± 67.72	160.83 ± 77.33	0.286
TG/HDL Ratio	3.61 ± 2.14	3.28 ± 1.88	0.114	3.38 ± 2.19	3.09 ± 1.69	0.116	3.76 ± 2.04	3.51 ± 2.32	0.181
Castelli I Index (TC/HDL)	4.13 ± 1.13	3.75 ± 0.79	0.002 *	3.93 ± 0.93	3.76 ± 0.85	0.066	4.33 ± 1.27	4.08 ± 0.97	0.134
Castelli II Index (LDL/HDL)	2.37 ± 0.88	2.09 ± 0.65	0.005 *	2.29 ± 0.81	2.14 ± 0.77	0.149	2.58 ± 1.15	2.39 ± 0.76	0.169
Homocysteine, mmol/L	9.91 ± 12.78	8.80 ± 2.84	0.477	9.12 ± 3.84	9.03 ± 2.55	0.873	10.36 ± 5.68	10.51 ± 6.38	0.214

^a^ Student’s *t* test, paired. * Statistically significant results. EVOO: extra virgin olive oil. DieTBra: traditional Brazilian diet. LDL-c: low-density lipoprotein cholesterol. BP: blood pressure. HOMA-IR: homeostatic model assessment of insulin resistance. HDL-c: high-density lipoprotein cholesterol. TG: triglycerides. TC: total cholesterol.

**Table 3:** Comparison of delta values and percentual difference from baseline of cardiometabolic risk factors in severely obese individuals after 12 weeks of follow-up.

Endpoints (12 Weeks–Baseline)	EVOO			DieTBra			DieTBra + EVOO		All Groups	
	Mean ± SD	95% CI	Percentual Difference from Baseline	Mean ± SD	95% CI	Percentual Difference from Baseline	Mean ± SD	95% CI	Percentual Difference from Baseline	*p* ^a^
∆ LDL-c	−5.11 ± 21.79	−11.82–1.59	−4.78	−4.27 ± 23.84	−11.79–3.26	−4.01	−1.93 ± 33.61	−11.92–8.05	−1.68	0.921
∆ Glycemia	−1.28 ± 18.27	−6.98–4.41	−1.22	−5.53 ± 23.48	−12.76–1.69	−5.15	−3.06 ± 31.29	−12.25–6.12	−2.59	0.668
∆ SBP	−0.53 ± 14.24	−4.92–3.85	−0.41	−2.51 ± 1.80	−6.16–1.13	−1.98	−3.64 ± 13.55	−7.62–0.34	−2.79	0.529
∆ BPB	−0.51 ± 9.43	−3.41–2.39	−0.59	−1.90 ± 8.42	−4.53–0.72	−2.25	−2.37 ± 8.20	−4.78–0.04	−2.75	0.693
∆ Insulinemia	−0.23 ± 16.69	−5.37–4.90	−0.91	0.67 ± 13.52	−3.49–4.83	2.74	0.98 ± 10.07	−1.97–3.94	4.75	0.809
∆ HbA1c	0.09 ± 0.87	−0.17–0.36	1.47	−0.32 ± 1.52	−0.79–0.15	−5.11	−0.02 ± 1.70	−0.53–0.47	−0.31	0.212
∆ HOMA-IR	−0.31 ± 5.39	−1.96–1.35	−4.57	−0.20 ± 3.83	−1.38–0.97	−2.99	0.54 ± 4.63	−0.83–1.92	9.39	0.709
∆ TC	−5.51 ± 30.44	−14.88–3.86	−2.93	−6.55 ± 28.39	−15.29–2.18	−3.55	−1.40 ± 36.66	−12.17–9.36	−0.72	0.686
∆ HDL-c	1.90 ± 8.17	−0.61–4.42	4.03	0.46 ± 8.90	−2.27–3.21	0.95	1.59 ± 8.35	−0.86–4.05	3.36	0.362
∆ Triglycerides	−6.67 ± 41.35	−19.4–6.05	−4.16	−14.56 ± 51.19 ^b^	−30.31–1.19	−9.41	−6.55 ± 41.59	−18.76–5.66	−3.95	0.717
∆ Non-HDL-c	−7.42 ± 26.13	−15.46–0.62	−5.34	−7.02 ± 25.77	−14.95–0.91	−5.15	−3.00 ± 35.57	−13.44–7.44	−2.02	0.724
∆ TG/HDL	−0.25 ± 1.03	−0.57–0.06	−3.61	−0.33 ± 1.35	−0.75–0.85	−9.38	−0.25 ± 1.29	−0.63–0.12	−6.65	0.975
∆ Castelli I	−0.33 ± 0.68	−0.54–−0.12	−7.99	−0.20 ± 0.69	−0.41–0.14	−5.09	−0.22 ± 0.99	−0.51–0.07	−5.08	0.550
∆ Castelli II	−0.26 ± 0.59 ^b^	−0.44–−0.08	−10.97	−0.14 ± 0.62	−0.33–0.05	−6.11	−0.18 ± 0.89	−0.45–0.08	−6.98	0.533
∆ Homocysteine	−1.48 ± 13.53 ^b^	−5.64–2.68	−14.93	−0.07 ± 3.02	−1.00–0.85	−0.77	0.33 ± 1.78	−0.19–0.85	3.18	0.992

^a^ Kruskal–Wallis. ^b^ Approximately 10% reduction. EVOO: extra virgin olive oil. DieTBra: traditional Brazilian diet. LDL-c: low-density lipoprotein cholesterol. BP: blood pressure. HOMA-IR: homeostatic model assessment of insulin resistance. HDL-c: high-density lipoprotein cholesterol. TG: triglycerides. TC: total cholesterol.

**Table 4:** Changes in caloric and macronutrient intake during the study period.

	EVOO			DieTBra			DieTBra + EVOO		
	Baseline	12 Weeks	*p* *	Baseline	12 Weeks	*p* *	Baseline	12 Weeks	*p* *
Calories	1615.33 ± 582.45	1733.00 ± 552.38	0.076	1687.9 ± 592.20	1267.47 ± 361.89	<0.001	1771.32 ± 905.06	1440.36 ± 391.26	0.002
Carbohydrates (%)	53.84 ± 6.12	48.10 ± 6.72	<0.001	53.16 ± 8.59	54.05 ± 6.86	0.483	52.04 ± 7.48	46.14 ± 6.36	<0.001
Proteins (%)	17.62 ± 3.63	16.52 ± 3.40	0.077	17.66 ± 4.56	19.27 ± 4.46	0.062	18.81 ± 4.71	17.59 ± 5.40	0.360
Total fat (%)	28.54 ± 5.11	35.37 ± 7.13	<0.001	29.17 ± 6.74	26.68 ± 5.55	0.032	29.15 ± 5.58	36.27 ± 7.45	<0.001
Saturated fat (g)	15.09 ± 7.35	14.21 ± 7.95	0.516	16.74 ± 7.68	9.39 ± 5.87	<0.001	16.13 ± 9.85	12.17 ± 5.42	0.002
MUFA (g)	15.25 ± 7.27	27.39 ± 11.30	<0.001	14.30 ± 7.54	9.26 ± 5.67	<0.001	15.79 ± 8.94	26.86 ± 12.70	<0.001
PUFA (g)	8.35 ± 4.14	8.73 ± 3.08	0.269	7.92 ± 4.41	5.27 ± 3.14	0.002	9.08 ± 5.47	7.68 ± 4.59	0.084

* Paired Student’s *t*-test. EVOO: extra virgin olive oil. DieTBra: traditional Brazilian diet. MUFA: monounsaturated fatty acids. PUFA: polyunsaturated fatty acids.

## Supplementary Materials

The following are available online at https://www.mdpi.com/2072-6643/12/5/1413/s1. Table S1: Main features of the Traditional Brazilian Diet (DieTBra) versus the Mediterranean Diet (MedDiet).
