# Supplementary material for: Traditional Brazilian Diet and Olive Oil Reduce Cardiometabolic Risk Factors in Severely Obese Individuals: A Randomized Trial
Source: Nutrients. 2020 May 14;12(5):1413. doi: 10.3390/nu12051413 (PMC7284483; doi:10.3390/nu12051413)
Supplement: Supplementary file 1 [file nutrients-12-01413-s001.pdf]

Supplementary data

Supplementary Table S1. Main features of the Traditional Brazilian Diet (DieTBra) *versus* the Mediterranean Diet (MedDiet).

**DieTBra**	**MedDiet**
• Plenty of tropical fruits within small meals, raw and cooked vegetables and legumes at lunch and dinner<br>• Bread, coffee and milk at breakfast<br>• Moderate consumption of dairy products (whole milk, yellow and white cheese, yogurt, curd, butter)<br>• Small portion of red meat<br>• Rare seafood<br>• Rare nuts<br>• Rice and beans at lunch and dinner<br>• Predominant use of soy oil to prepare meals	• Plenty of vegetables, legumes, seeds<br>• Whole grains<br>• Breads<br>• Moderate consumption of poultry, eggs, cheese and yogurt<br>• Rare consumption of red meat<br>• Seafood<br>• Nuts<br>• Great amount of olive oil<br>• Wine
